# Validation of multi-stage telephone-based identification of cognitive impairment and dementia

**DOI:** 10.1186/1471-2377-5-8

**Published:** 2005-04-13

**Authors:** Valerie C Crooks, Linda Clark, Diana B Petitti, Helena Chui, Vicki Chiu

**Affiliations:** 1 Department of Research & Evaluation, Kaiser Permanente Southern California, 100 S. Los Robles, Pasadena, CA91101, USA; 2 Alzheimer's Disease Research Center, University of Southern California, 3715 McClintock Ave., Los Angeles, CA90089-0191, USA

## Abstract

**Background:**

Many types of research on dementia and cognitive impairment require large sample sizes. Detailed in-person assessment using batteries of neuropyschologic testing is expensive. This study evaluates whether a brief telephone cognitive assessment strategy can reliably classify cognitive status when compared to an in-person "gold-standard" clinical assessment.

**Methods:**

The gold standard assessment of cognitive status was conducted at the University of Southern California Alzheimer Disease Research Center (USC ADRC). It involved an examination of patients with a memory complaint by a neurologist or psychiatrist specializing in cognitive disorders and administration of a battery of neuropsychologic tests. The method being evaluated was a multi-staged assessment using the Telephone Interview of Cognitive Status-modified (TICSm) with patients and the Telephone Dementia Questionnaire (TDQ) with a proxy. Elderly male and female patients who had received the gold standard in-person assessment were asked to also undergo the telephone assessment. The unweighted kappa statistic was calculated to compare the gold standard and the multistage telephone assessment methods. Sensitivity for classification with dementia and specificity for classification as normal were also calculated.

**Results:**

Of 50 patients who underwent the gold standard assessment and were referred for telephone assessment, 38 (76%) completed the TICS. The mean age was 78.1 years and 26 (68%) were female. When comparing the gold standard assessment and the telephone method for classifying subjects as having dementia or no dementia, the sensitivity of the telephone method was 0.83 (95% confidence interval 0.36, 1.00), the specificity was 1.00 (95% confidence interval 0.89,1.00). Kappa was 0.89 (95% confidence interval 0.69, 1.000). Considering a gold-standard assessment of age-associated memory impairment as cognitive impairment, the sensitivity of the telephone approach is 0.38 (95% confidence interval 0.09, 0.76) specificity 0.96 (CI 0.45, 0.89) and kappa 0.61 (CI 0.37, 0.85).

**Conclusion:**

Use of a telephone interview to identify people with dementia or cognitive impairment is a promising and relatively inexpensive strategy for identifying potential participants in intervention and clinical research studies and for classifying subjects in epidemiologic studies.

## Background

Epidemiologic studies of dementia generally require a large number of subjects. Longitudinal study designs that start with subjects who are cognitively intact and follow them to ascertain the development of dementia are often needed to reduce bias. Although physician and neuropsychological evaluations are considered the gold standard for the diagnosis of dementia, the high cost of these methods makes them infeasible for population-based epidemiological surveys. Administration of mental status screening tests and functional questionnaires by telephone offers a less costly alternative. These methods minimize participant burden, permit standardization and use less costly personnel. However, few studies have examined the reliability and validity of telephone-based methods in correctly identifying cognitive impairment and dementia [1].

This study evaluates whether a brief multi-stage telephone-based cognitive test and functional questionnaire strategy can reliably classify cognitive status when compared to a gold standard in-person clinical assessment. In this study, the gold standard was a classification derived by in-person neurological and neuropsychological evaluation at a university-based Alzheimer Disease Research Center. The staged telephone assessment of cognitive status has been used in the Kaiser Permanente Women's Memory Study, a large ongoing epidemiologic study of dementia in a sample of 3,681 women 75 and older. The same staged model was used in the veteran twin study by Gallo and Breitner [2] and a similar staged model for assessment of dementia and cognitive impairment was used in the Cache County study [3].

## Methods

### Overview

The study compared the classification of elderly patients into 3 categories (dementia, cognitive impairment or cognitively unimpaired) using two different assessment approaches. The gold standard reference classification was performed at the University of Southern California Alzheimer Disease Research Center (USC ADRC) and involved clinical examination of patients by a neurologist and a team experienced in the diagnosis of dementia and cognitive impairment. The second approach relied on information from telephone interviews, the Telephone Interview of Cognitive Status-modified (TICSm) [4,5], and a computer-assisted telephone version of the Dementia Questionnaire [6]. In this study we refer to this interview as the Telephone Dementia Questionnaire (TDQ). The comparison study was reviewed and approved by the Kaiser Permanente Southern California and University of Southern California Institutional Review Boards (IRBs).

### Subjects and procedures

The subjects in this study were enrolled in a longitudinal study of normal aging, cognitive impairment, and Alzheimer-type dementia at the University of Southern California (USC) Alzheimer Disease Research Center (ADRC). Initial evaluations involve clinical examination of patients by a Board-certified neurologist and a licensed neuropsychologist experienced in the assessment and diagnosis of dementia. The neuropsychological battery was comprised of 20 tests of memory, language, executive control, and construction. In addition, scales of mood and psychiatric symptoms and screenings for visual or auditory impairment were administered to the subject. A rating of functional ability was provided by a collateral informant. See Appendix I for a list of the tests given.

Patients evaluated at the USC ADRC are classified as possible or probable AD, mild cognitive impairment (MCI), age-associated memory impairment (AAMI), or normal [7]. One patient in this study had a diagnosis of mixed dementia because she had AD and a history of stroke. Table 1 shows the criteria used to classify patients in each of these four categories.

**Table 1:** Diagnostic criteria for Alzheimer disease and cognitive impairment

Probable Alzheimer disease	By criteria of the National Institute of Neurological and Communicative Disorders and Stroke and the Alzheimer's Disease and Related Disorders Association (NINCDS-ADRDA) [6] Dementia by clinical examination, and documented with the Mini-Mental Status Exam (MMSE) and the Short-Blessed functional impairment questionnaire, with progressive deficits in 2 or more cognitive domains. • Absence of other disorders that could account for the progressive dementia symptoms
Possible Alzheimer disease	By NINCDS-ADRDA criteria : • Dementia, as defined above; • Presence of 1) variations in the onset, presentation or clinical course; - or - 2) other disorder(s) sufficient to produce dementia, but not thought to be the cause of the dementia in this case

Mild Cognitive Impairment	• Subjective memory complaint • Normal general cognitive function by MMSE • Standard score of -1.5, below the same-age norm, on the long delay free recall item of the California Verbal Learning Test (CVLT)

Age-Associated Memory Impairment	• Has both Subjective memory complaint and normal general cognitive function by MMSE • And either: 1) Standard score of -1.0, below the norm for 20–24 year- olds, on the long delay free recall item of the CVLT - or - 2) Clinical Dementia Rating score of 0.5 - or - 3) Significant decline from previous performance on cognitive test(s) while still scoring in normal range

Follow-up evaluation is comprised of an interval history, depression, behavior and functional questionnaire and neuropsychological testing. Follow-up evaluation is conducted annually for subjects who are classified as cognitively impaired, demented or cognitively intact but over age 80 years and is repeated every 2 years for normal controls less than 80 years old. The results of the follow-up evaluation and neuropsychological testing are reviewed by a team of neurologists and psychologists and the diagnostic classification is updated accordingly. For this study, the most recent classification was used.

Ninety-eight subjects (representing normal, cognitively impaired and dementia cases) at the USC ADRC during the past 2 years were invited to participate in this study. They were contacted by letter by the USC ADRC (with a return opt-in postcard) to obtain initial permission to contact for a possible telephone interview. Postcard or verbal permission to contact was obtained in 50 cases.

Kaiser Permanente investigators mailed an informed consent form to the subject or proxy. Shortly thereafter, a telephone contact was made. Participants read and discussed the consent with the research assistant and were instructed to sign and return the written consent form by mail. If consent was obtained, telephone contact information for these participants (and their consenting proxies for patients with dementia) and appointment times were provided to the survey research firm that conducts the telephone cognitive assessments.

Interview responses were reviewed using the procedures described below. Both the telephone interviewers and the experts making the classification of cognitive status based on the TDQ were blinded to the results of the USC ADRC assessment until all subjects in the study had been classified.

### Women's Memory Study assessment of dementia and cognitive status

The first step in the Women's Memory Study assessment of dementia and cognitive status is administration of the 23-question TICSm, which takes from 8 to 15 minutes to administer. The TICSm is strongly correlated with the Mini-Mental State Exam [8,5,10], a more widely used brief cognitive screen. The TICSm has been previously validated [4] and the psychometric properties of the computer-assisted TICSm used in the Women's Memory Study has also been evaluated [11]. Prior studies have shown that the TICSm has a high sensitivity in the detection of dementia [2,12] but a low positive predictive value [4]. Similar to an earlier study [2] TICsm cut-off scores are used to classify individuals as having no cognitive impairment (TICS score > 27) or possible cognitive impairment (TICSm score ≤ 27).

For those classified as possibly impaired based on the TICSm score, an attempt is made to do a second stage cognitive assessment in which the Telephone Dementia Questionnaire (TDQ), is administered to a proxy. The TDQ is a previously validated instrument [13,14] that takes about 19 minutes to complete and asks the proxy up to 48 questions about the subject's cognitive function in several domains (memory, fluency, comprehension, orientation). The TDQ alone, when compared to antemortem clinical exams, was found to have 92.8% sensitivity for dementia and a specificity of 89.5% [15]. In previous work by Gallo and Breitner [2] when used with the TICSm, the specificity of cognitive classification was .99. Two trained assessors independently review the TDQ responses and use this information and pre-defined criteria to classify individuals in one of three categories: 1) definite dementia; 2) no or minimal cognitive impairment; or 3) cognitive impairment without definite evidence of dementia.

TDQ classification in the dementia category requires memory deficits and multiple impairments in another cognitive domain and at least one deficit in an additional cognitive domain. Classification in the category of "no or minimal impairment" requires no more than two "yes" responses to questions about problems with memory. Classification as cognitive impairment without definite evidence of dementia requires a scatter of deficits that were not sufficient for a dementia classification but were too many to be classified as no dementia. Overall, classification of dementia based on the two-stage telephone classification is "conservative" in that it requires multiple impairments in memory plus 2 additional cognitive domains and functional impairment.

The reviewers then make a consensus TDQ classification in the same three categories after discussion of the independent assessments. When there is agreement among the reviewers, no further review is done. When the reviewers disagree, a third trained reviewer, a neuropsychologist or a neurologist, assesses the TDQ responses and makes a final classification. The test reliability of the consensus TDQ has been measured on an on-going basis by having the reviewers reassess TDQs selected at random and blinded to the initial assessment. The kappa coefficient was 0.85 for the consensus assessments.

### Analysis

The analysis compared the USC ADRC final diagnosis of cognitive status and the Women's Memory Study classification based on telephone surveys. The unweighted kappa statistic was calculated comparing the ADRC and the staged telephone classification. As this was the principal aim of the Women's Memory Study method, the first comparison included dementia and no dementia classifications only.

Other comparisons were based on two alternate assumptions about patients who had been classified by the USC ADRC as having Age-Associated Memory Impairment (AAMI). The first assumption was that these patients were normal and the second was that they had cognitive impairment. Sensitivity for classification with dementia and specificity for classification as normal were also calculated under both sets of assumptions. Ninety-five percent confidence intervals for kappa were estimated as described in Fleiss [16]. Confidence intervals for sensitivity and specificity are exact intervals calculated using StatXact 5 software [17].

## Results

Fifty patients who completed the USC ADRC evaluations initially consented to participate in this study. We were able to complete the TICSm assessments on 38 (76%) of these patients. The mean age of participants was 78.1 years (SD ± 8.0) and 26 (68%) were female. Eight of the 38 patients scored below 28 on the TICSm. We then completed TDQ interviews for 8 of the 8 proxies (100%).

Table 2 shows the final USC ADRC diagnoses in four ADRC categories and the telephone survey classifications in three categories for all 38 cases.

**Table 2:** Comparison of results of USC ADRC and telephone survey-based classification.

		USC ADRC Classification				
		
		AD or Mixed Dementia	MCI	AAMI	Normal	All
Classification by Telephone Assessment	Dementia	5	0	0	0	5
	Cognitive Impairment	0	2	1	1	4
	No or Minimal Impairment	1	2	3	23	29
	All	6	4	4	24	38

AD Alzheimer's disease

MCI mild cognitive impairment

AAMI age-associated memory impairment

Table 3 shows the classifications broken down into two categories for both the USC ADRC and telephone classifications: dementia and not dementia. When using this staged classification method, the sensitivity of the telephone method to detect dementia was 0.83 (95% CI 0.36,1.00), and the specificity to classify as not dementia was 1.00 (95% CI 0.89,1.00). Kappa was 0.89 (95% CI 0.69,1.00).

**Table 3:** Comparison of results of USC ADRC "gold standard" and telephone survey-based classification of dementia and no dementia

		Gold Standard Classification		
		
		Dementia	Not Dementia	Total
Classification by Telephone Assessment	Dementia	5	0	5
	Not Dementia	1	32	33
	All	6	32	38

Telephone Assessment

Sensitivity for dementia 5/6 = 0.83 (95% confidence interval 0.36, 1.00)

Specificity for not dementia 32/32 = 1.00 (95% confidence interval 0.89, 1.00)

Kappa 0.89 (95% confidence interval 0.69, 1.00)

Tables 4 and 5 show both the ADRC and telephone classifications in three categories under two sets of assumptions about whether individuals with AAMI are properly classified as normal or cognitively impaired. In both tables, whether AAMI is classified as normal or cognitively impaired, sensitivity to detect dementia is 0.83 (95% C.I. 0.35, 0.99). In Table 4 under the assumption that AAMI is normal, the specificity of the telephone approach is 0.93 (95% C.I. 0.77, 0.99), while the sensitivity for cognitive impairment is 0.50 (95% C.I. 0.07, 0.93). Kappa is 0.67 (95% C.I. 0.45, 0.89). In Table 5 under the assumption that AAMI is cognitive impairment, the specificity of the telephone classification is 0.96 (95% C.I. 0.79, 1.00) and the sensitivity for cognitive impairment is 0.38 (95% C.I. 0.09, 0.76). Kappa is 0.61 (95% C.I. 0.37, 0.85).

**Table 4:** Comparison of results of USC ADRC and telephone survey-based classification in three categories. Assumption 1: AAMI (age-associated memory impairment) considered normal.

		Gold Standard Classification			
		
		AD or Mixed Dementia	Cognitive Impairment	Normal	All
Classification by Telephone Assessment	Dementia	5	0	0	5
	Cognitive Impairment	0	2	2	4
	No or Minimal Impairment	1	2	26	29
	All	6	4	28	38

Telephone Assessment

Sensitivity for dementia 5/6 = 0.83 (95% confidence interval 0.35, 0.99)

Sensitivity for cognitive impairment 2/4 = 0.50 (95% confidence interval 0.07, 0.93)

Specificity for normal 26/28 = 0.93 (95% confidence interval 0.77, 0.99)

Kappa 0.67 (95% confidence interval 0.45, 0.89)

**Table 5:** Comparison of results of USC ADRC and telephone survey-based classification in three categories. Assumption 2: AAMI (age-associated memory impairment) considered cognitive impairment.

		Gold Standard Classification			
		
		AD or Mixed Dementia	Cognitive Impairment	Normal	All
Classification by Telephone Assessment	Dementia	5	0	0	5
	Cognitive Impairment	0	3	1	4
	No or Minimal Impairment	1	5	23	29
	All	6	8	24	38

Telephone Assessment

Sensitivity for dementia 5/6 = 0.83 (95% confidence interval 0.35, 0.99)

Sensitivity for cognitive impairment 3/8 = 0.38 (95% confidence interval 0.09, 0.76)

Specificity for normal 23/24 = 0.96 (95% confidence interval 0.79, 1.00)

Kappa 0.61 (95% confidence interval 0.37, 0.85)

Table 6 gives the TICSm scores and other clinical information for patients with definite disagreement between the ADRC and the telephone classification. These included the single patient classified as having dementia by the ADRC and as unimpaired based on the telephone assessment; the two patients classified as having no or minimal impairment in the telephone assessment and Mild Cognitive Impairment (MCI) by the ADRC; and the one patient classified with cognitive impairment in the telephone assessment who was classified as normal by the USC ADRC.

**Table 6:** TlCSm scores and other clinical information for patients with definite disagreement between USC ADRC classification and telephone survey classification.

USC ADRC Classification	Telephone Survey Classification	TICSm Score	Clinical History
Dementia	No/minimal impairment	29	--
MCI	No/minimal impairment	28	History of seizures
MCI	No/minimal impairment	30	History of cardiovascular disease
Normal	Cognitive impairment	24	Anxiety and depression

MCI mild cognitive impairment

## Discussion

Our study shows that assessment of dementia status in elderly subjects by a multi-stage telephone method has good agreement with a comprehensive in-person dementia assessment. Sensitivity and specificity for classification of cognitively normal or no dementia were also good. The one case of dementia classified falsely as not impaired in the telephone assessment may have been due to the high educational level (BA degree) and IQ (estimated premorbid Verbal IQ of 120) of this subject. Our approach does not make adjustments for education in the TICSm cutpoint, therefore dementia in persons with high premorbid education and/or IQ may be missed.

Note that dementia in this study was confined to AD dementia and the definition of dementia required multiple problems with memory. This study does not specifically address the accuracy of the telephone methods for detecting other types of dementia which are not characterized by predominant memory impairment.

On the other hand, sensitivity and specificity of the telephone approach for cognitive impairment was low, especially if one considers AAMI as a form of cognitive impairment. Even in clinical settings, however, the criteria for measuring and defining mild cognitive impairment (MCI) are not uniform or standardized [18,19]. Consensus on the definition of MCI would be critical for clinicians and researchers since a number of studies have suggested that MCI is a prodrome or precursor to dementia. Progression or conversion from MCI to dementia can range from 6–25% annually [20,21] to 23 – 47% over 2.6 years [22]. Clinic-based studies of MCI suggest more uniform progression to dementia than population-based ones where the classification is more unstable [23]. Regardless of assessment method used, the instability of the MCI classification can be seen in studies where one third [24,25] to one half revert back to normal cognition [26]. These findings could explain some of the classification differences between our two assessment methods.

Reliance on the telephone assessment would misclassify some subjects. In general, misclassification in epidemiologic studies causes a bias to the null when it is non-differential [27]. In large population studies, because screening test scores often detect possible dementia or cognitive impairment, the percentage of subjects invited for in-person clinical and neuropsychologic examinations can be substantial. Failure to participate in clinical examinations also has the potential to cause bias especially if failure to attend is related to cognitive impairment. In the Cache County study, for example, 31% of subjects with an indication of dementia did not return for the requested clinical work-up [3]. In comparing in-person assessment of cognitive status using gold-standard neuropyschologic testing with telephone assessment, bias due to misclassification must be balanced against bias due to non-response.

Epidemiologic studies that involve in-person assessments of cognition are costly compared with those that rely solely on telephone assessment. We estimate that the telephone strategy used here costs about $38.00 per person assessed when the percentage of those assessed who "fail" the TICSm (score ≤ 27) is 25%. The costs of in-person assessments are generally 20 times that cost.

The costs of in-person assessments also affected the number of cases in this analysis. The small number of patients who were willing to be interviewed for the in-person assessments as well as follow-up telephone interviews is a further limitation. In our previous work we have found that there tends to be a bias toward non-participation for the cognitively impaired and demented [28].

## Conclusion

Use of the multi-stage telephone strategy to identify people who have dementia or cognitive impairment is promising as a way to identify potential participants in intervention or clinical research. Use of the strategy in dementia detection studies is less costly, does not require representative samples, can be generally smaller than epidemiologic studies, and can be designed to validate eligibility prior to study enrollment.

## Competing interests

None of the authors have competing personal or financial interests in the interpretation or presentation of this data.

## Authors' contributions

VCC & DBP contributed to all aspects of design, analyses and implementation and interpretation of study, and drafts, revisions and critical review of paper.

LC & HC contributed to implementation of study, interpretation of data and critical review of paper.

VC contributed to analyses and critical review of paper.

All have given final approval of this submission.

## Appendix I. USC ADRC Test Battery

Screening

Mini Mental State Examination

Short Blessed Test

Estimated Premorbid IQ

American Version of the Nelson Adult Reading Test (AMNART)

Language

Boston Naming Test

Phrase repetition – Boston Diagnostic Aphasia Exam

Oral Word Association Test

Animal Naming

Token Test – Multilingual Aphasia Examination

Construction

Rey-Osterrieth Complex Figure, copy

Judgment of Line Orientation

Clock Drawing

Block Design -WAIS- R

Memory

Logical Memory I and II- Wechsler Memory Scale III

California Verbal Learning Test – II

Faces I and II – Wechsler Memory Scale III

Rey-Osterrieth Complex Figure 3' Delay

Executive Control

Digit Span, forward and backward – WAIS-R

Trail Making, A and B

Number-Letter Sequencing – Wechsler Memory Scale III

Repeated patterns

Emotional Status

Geriatric Depression Scale

Symptoms Checklist 90-R

Functional Ability

Clinical Dementia Rating Scale (completed by a collateral informant)

## Pre-publication history

The pre-publication history for this paper can be accessed here:


